# B7‐H3 promotes nasopharyngeal carcinoma progression by regulating CD8+ T cell exhaustion

**DOI:** 10.1002/iid3.70005

**Published:** 2024-09-13

**Authors:** Zhaoen Ma, Gui Chen, Hao Li, Saixuan Yang, Yali Xu, Bolin Pan, Wuping Lai, Guangui Chen, Wenjing Liao, Xiaowen Zhang

**Affiliations:** ^1^ The First Affiliated Hospital of Jinan University Guangzhou China; ^2^ Department of Otolaryngology The Second Affiliated Hospital of Guangzhou Medical University Guangzhou China; ^3^ Department of Otolaryngology, Head and Neck Surgery The First Affiliated Hospital of Guangzhou Medical University Guangzhou China; ^4^ Guangzhou Medical University Guangzhou China

**Keywords:** 4‐1BB, B7‐H3, CD8+ T, nasopharyngeal carcinoma, tumor immunity

## Abstract

**Background:**

B7‐H3 protein is an important regulator of the adaptive immune response in human tumorigenesis. 4‐1BB is a co‐stimulatory receptor expressed on activated CD8+ T cells, and regulates T cell immunity. Here, we investigated the role of B7‐H3 in the growth and invasion of nasopharyngeal carcinoma (NPC) and the effect of its interaction with 4‐1BB on tumor immunity.

**Methods:**

Short hairpin (sh) RNA was designed to knock down B7‐H3 expression in NPC cells. NPC cells with stable knockdown of B7‐H3 were established and injected into nude mice. The effects of B7‐H3 on cell proliferation, apoptosis, and epithelial‐to‐mesenchymal transition (EMT) were detected by the CCK8 assay, flow cytometry, TUNEL assay, and western blot analysis. The migration and invasion abilities were determined using the Transwell assay and scratch assay. Co‐immunoprecipitation (Co‐IP) assays were performed to study the interaction between B7‐H3 and 4‐1BB. Anti‐4‐1BB antibody was used in a co‐culture system and xenograft mice to study the effect of 4‐1BB on NPC development.

**Results:**

NPC cells transfected with sh‐B7‐H3 showed a higher rate of apoptosis, slower growth rate, impaired migration, and less EMT in vitro. Xenograft mice with stable knockout of B7‐H3 had lower tumor burdens, and the stripped tumors had lower rates of cell proliferation, higher rates of apoptosis, and less EMT in vivo. Additionally, decreased B7‐H3 expression was positively correlated with interferon‐γ, tumor necrosis factor‐α, and 4‐1BB+CD8+ tumor‐infiltrating lymphocytes. Co‐IP studies showed that B7‐H3 interacts with 4‐1BB. Also, the inhibitory effects of sh‐B7‐H3 on NPC tumor growth, invasion, and tumor immunity could be alleviated by the anti‐4‐1BB antibody both in vivo and in vitro.

**Conclusion:**

Our findings suggest that B7‐H3 may accelerate tumor growth, tumor cell invasion, and EMT, and interact with 4‐1BB to produce CD8+ T cell exhaustion that inhibits tumor immunity. B7‐H3 might serve as a novel target for treating NPC.

## 1 INTRODUCTION

Nasopharyngeal carcinoma (NPC) is one of the most common malignant tumors of the head and neck. It originates from epithelial cells in the inner lining of the nasopharyngeal mucosa, and is closely correlated with Epstein‐Barr virus (EBV) infection. NPC is prevalent in South China, South‐Eastern Asia, and North Africa, and has unique ethnic and geographic distributions.^1^

B7‐H3, also known as CD276, is an important regulator of the adaptive immune response and a key factor in human tumorigenesis. B7‐H3 is rarely expressed and present only at low levels in normal human tissues.^2^ However, recent studies have found abnormally high expression of B7‐H3 in many common malignancies, including malignant tumors of the stomach, lungs, prostate, kidneys, ovaries, and endometrium. Furthermore, a high level of B7‐H3 expression affects a patient's prognosis.^3^ Interestingly, some quantitative PCR (qPCR) studies suggest that CD276 mRNA expression in NPC tissues is higher than that in non‐NPC tissues, and gene‐expression profiling from GEO (GDS3341, GSE40290, and GSE5381968) has revealed that the levels of CD276 mRNA in NPC tissues are significantly higher than those in non‐NPC tissues.^4^

The conversion and establishment of a latent EBV infection is an important step in the pathogenesis of NPC, and type II latency is observed in NPC.^5^ In addition to EBER and EBNA1, LMP1 and LMP2 have also been confirmed in EBV‐infected NPC. B7‐H3, which EBV can upregulate, has been found to inhibit natural killer (NK) cell‐mediated antitumor function and promote NPC progression, suggesting its association with EBV latent procedures.^6^ Previous work has shown that B7‐H3 is co‐stimulatory for T cells,^7^ and can selectively stimulate interferon‐γ (IFN‐γ) production and enhance the induction of primary human CD8+ cytotoxic T‐cells (CTLs).^8^ Additionally, in several mouse cancer models, ectopic expression of B7‐H3 was shown to lead to activation of tumor‐specific CTLs, which are able to slow tumor growth or even completely eradicate tumors. Other studies have identified B7‐H3 in ovarian tumor vessels and found it was associated with adverse clinical outcomes, indicating that tumors may exploit B7‐H3 to downregulate antitumor immunity via T cells. Therefore, tumor‐associated B7‐H3 offers a new therapeutic opportunity for enhancing antitumor immunity or might serve as a drug target.

According to SRTING, B7‐H3 is predicted to interact with tumor necrosis factor receptor superfamily 9 (TNFRSF9), also known as CD137 or 4‐1BB, which was first discovered in the 1980s in activated T cells. It was found that activation of 4‐1BB on the surface of CAR‐T cells loaded with B7‐H3 antibody significantly enhanced their antitumor effect and reduced the level of PD‐1 expression.^9^ B7‐H3 has shown promise as a therapeutic target for immunotherapy; however, its role in NPC is not well understood due to a lack of studies in that area. Thus, our study investigated whether B7‐H3, which is highly expressed by NPC tumor cells, interacts with 4‐1BB protein on the surface of CD8+ T cells to inhibit its protein activity and ultimately suppress the tumor antigen‐specific immune response.

Our findings suggest that the inhibitory effect of B7‐H3, a protein which is expressed at high levels in NPC tumor cells, on the immune response against tumor cells can be attributed to its interaction with 4‐1BB, which is a protein located on the surface of CD8+ T cells. This interaction results in the suppression of 4‐1BB protein activity.

## 2 METHODS

### 2.1 Cell culture

Human NPC cell lines, including CNE2 cells and highly metastatic NPC cells (5‐8F), were purchased from ATCC and frozen in liquid nitrogen after expansion for 3–5 passages. The initial revival cells from frozen stocks were passaged 5–10 times before use for in vitro or in vivo experiments. The cells were cultured in DMEM high glucose medium (Gibco, Invitrogen) supplemented with antibiotics–antimycotic (Gibco) and 10% fetal bovine serum (FBS) (Gibco) at 37°C in a humidified atmosphere of 95% air and 5% CO_2_.

### 2.2 Cell transfection and generation of a stable cell line

B7‐H3 suppression was achieved by using shRNA targeting a specific sequence in the exon of B7‐H3. A negative control shRNA and specific shRNAs targeting B7‐H3 were designed and acquired from Synbio Technologies, and their sequences are shown in Table 1. Approximately 2 × 10^5^ cells were seeded into each well of a six‐well cell culture plate and cultured overnight before transfection. A mixture containing 2 μg of sh‐B7‐H3 or sh‐NC and 5 μL of Lipofectamine™ 3000 was added to 250 μL of serum‐free medium, and the solutions were mixed to generate a shRNA/Lipofectamine™ 3000 complex, which was added to the target cells and incubated for 4–6 h. Next, the transfection solution was removed and the cells were cultured in complete culture medium for 48 h before being harvested and analyzed by real‐time qPCR (RT‐qPCR) to select the most efficient sh‐B7‐H3‐RNA sequence.

**Table 1:** The sequences of specific shRNAs targeting B7‐H3.

Target name	Sequence (5′–3′)
h*B7‐H3‐1	CAAAGAAGATGATGGACAAGA
h*B7‐H3‐2	TGGTGCACAGTTTCACCGAAG
h*B7‐H3‐3	CAACGAGCAGGGCTTGTTTGA

To establish an NPC cell line with stable B7‐H3 knockdown, the culture medium was changed after 48 h of transfection, and replaced with a medium (screening medium) containing 600 μg/mL Puromycin (MedChemExpress). The cells were then cultured at 37°C with 5% CO_2_ for 72 h, with the medium being changed every 2 days. Next, the live cells were inoculated into 24‐well plates according to a limited dilution method to obtain a stable monoclonal cell line, which was subsequently cultured in the medium containing Puromycin (300 µg/mL) for 28 days. Western blot analysis was performed to verify the downregulation of B7‐H3 protein expression when screening for stable cell lines. These stable cell lines were then used in subsequent experiments.

### 2.3 Construction of a Luc stable cell line

The numbers CNE2 cells with stable B7‐H3 knockdown and the numbers of control cells in the logarithmic growth phase were counted, and their densities were adjusted to 1 × 10^5^/cells/mL. Next, 2 mL of cell suspension was added to a six‐well plate. Concentrated CMV‐luc‐Neo lentivirus (GenePharma) was added to infect the CNE2 cells with a complex (MOI = 10); after which, 1 mL of polybrene was added to a final concentration of 8 μg/mL. After 72 h of infection, G418 antibiotic medium (G418 concentration, 1 mg/mL) was added to the cells, and the medium was changed every 2 days. G418 screening was performed until monoclonal cells were obtained. Next, 1 × 10^5^ cells were collected from each well and seeded into a 24‐well plate. After 24 h of culture, passive lysis (YBscience, #YB‐191208) was used to lyse the cells. The lysed cells were centrifuged at 1200 rpm for 10 min at 4°C. Next, 10 μL of lysate was obtained and added to each well of a 96‐well plate, and 50 μL of luciferase substrate was added to each well and incubated for 2 s. The fluorescence value (RLU) at 10 s was recorded, and three multiple pores were set up in each clone. The cell clones with a high RLU value were retained for further culture. After five passages, luciferase activity was detected again, and the cell clones with a high RLU value continued to be cultured until the 30th generation. CNE2‐luc clones with the highest luciferase activity were retained for use in subsequent experiments.

### 2.4 Cell clonal formation experiment

Cells in a logarithmic growth phase from each group were digested and suspended in complete medium. The cell suspensions were diluted in gradient multiples and 100 cells were inoculated into a 3.5 cm dish containing 2 mL of pre‐heated culture medium at 37°C. The dish for each group was gently shaken to ensure an even cell suspension, and the cells were then cultured for 2–3 weeks. The culture was terminated when visible clones appeared in the Petri dish. The culture supernatants were discarded and the plates were washed twice with phosphate buffered saline (PBS). The Petri dishes were then washed with 5 mL of pure methanol and fixed for 15 min. After removing the fixation buffer, an appropriate amount of Giemsa dye solution was added to the dishes, and the dishes were let sit for 10–30 min. Next, the dye solution was washed away with running water, the dishes were dried with air, and the numbers of cell clones were counted under a microscope (low power lens).

### 2.5 Coculture experiments

Human NPC tissues were obtained and used to create single‐cell suspensions that were used to sort out CD8+ TILs by use of CD8+ beads (Miltenyi, 130‐121‐560). All NPC patients were recruited according to the protocol that was approved by the clinical Ethics Committees of the First Affiliated Hospital of Guangzhou Medical University. This study adhered to the tenets of the Declaration of Helsinki. Written informed consent was obtained from all participants. A 2 mL volume of CNE2 cell suspension was seeded into each well of a six‐well plate at a concentration of 1 × 10^5^/cells mL, and the cells were transfected as previously described. Next, 2 µg/mL of anti‐4‐1BB or anti‐IgG (Abcam) was added to the CD8+ TILs. After resuspension, the transfected CNE2 cells were mixed with the TILs in each group at a density ratio of 1:1, followed by co‐culture for 24 h. The effects of blocking 4‐1BB on the apoptosis of tumor cells and ratio of residual T cells were measured by flow cytometry (CytoFLEX S, Beckman Coulter).

### 2.6 Co‐immunoprecipitation (co‐IP) assays

Co‐IP assays were conducted using a Co‐IP Kit (Thermo, Pierce®, #26149) according to the manufacturer's instructions. Briefly, PMSF buffer containing a mixture of protease inhibitors was used to prepare protein lysates from the CNE2 cell plus CNE2 cell co‐culture systems. A spin column loaded with resin slurry was incubated with 4‐1BB antibody (Abcam, #ab270527) or B7‐H3 (Abcam, #ab134161) antibody; Rabbit IgG antibody (Proteintech, #30000‐0‐AP) served as a negative control. The protein lysates were incubated with the antibody‐loaded resin to obtain the target protein, which was then washed off with elution buffer. The eluted proteins were separated by sodium dodecyl sulfate polyacrylamide gel electrophoresis (SDS‐PAGE) and subsequently detected by western blot analysis.

### 2.7 Western blot analysis

The treated cells or tissues were harvested in RIRA buffer (Beyotime) and the protein concentration in each lysate was determined using the BCA assay. Next, an equal amount of total protein from each sample was separated by SDS‐PAGE, and the protein bands were transferred onto PVDF membranes, which were subsequently blocked with 5% skimmed milk for 1 h at room temperature. The PVDF membranes were then washed with TBST (containing NaCl, Tris‐HCl, and Tween‐20) and incubated with primary antibodies, including anti‐B7H3 (Abcam, #ab227670, 1:400), anti‐N‐cadherin (Abcam, #ab18203, 1:1000), anti‐E‐cadherin (Abcam, #ab40772, 1:1000), anti‐Bcl‐2 (Abcam, #ab182858, 1:2000), anti‐Bax (Abcam, #ab182733, 1:2000), and GAPDH (Abcam, #ab181602, 1:10,000) overnight at 4°C, followed by two TBST washes. The membranes were then incubated with a species‐specific horseradish peroxidase‐conjugated secondary antibody (HRP Goat anti‐Rabbit IgG, #abcam, ab6721, 1:2000) for 1 h at room temperature and washed three times with TBST. The immunostained proteins were visualized by chemiluminescence (ECL). GAPDH served as an internal control protein.

### 2.8 RT‐qPCR

RNA was extracted from cells using Trizol reagent (TaKaRa), and reverse transcribed into cDNA using a PrimeScript RT reagent kit with gDNA eraser (Takara). Quantitative PCR was performed on a LightCycler 96 real‐time PCR system (Bio‐Rad).

TB Green premix Ex Taq II was used in the experiments according to recommended guidelines (TaKaRa, #RR820). The melting curves and values were assessed using Bio‐Rad software. B7‐H3 expression was quantified by the $2^{-\Delta \Delta C_{T}}$ method with human GAPDH serving as an internal standard. Sequences of primers are shown in Table 2.

**Table 2:** The primers used for RT‐qPCR assay.

Gene	5′–3′
B7‐H3‐F	CTGAGGCTGAGGTGTTCT
B7‐H3‐R	GCAATGAGACAGACAGACA
GAPDH‐F	GGAGCGAGATCCCTCCAAAAT
GAPDH‐R	GGCTGTTGTCATACTTCTCATGG

### 2.9 Transwell assays

Matrigel® Matrix (Corning Costar) and Transwell chambers with 8‐μm apertures (Corning Costar) were used to perform Transwell assays. For invasion experiments, a layer of Matrigel was first applied to the bottom of the upper Transwell chamber. Next, a mixture of serum‐free medium and Matrigel at a ratio of 4:1 was prepared, and 100 μL of the diluted Matrigel® Matrix was added to the center of the upper chamber.

The obtained cells were resuspended in serum‐free medium at a concentration of 1 × 10^5^ cells/mL. Once the cell suspension (100 μL) was added to the upper Transwell chamber, 700 μL of complete medium with 10% FBS was added to the lower chamber. After a 48‐h incubation, the chamber was removed, and cells that had passed through the filtration membrane were fixed, stained, and counted under a microscope. The cell counts were conducted using ImageJ software.

### 2.10 Scratch assay

CNE‐2 and 5‐8F cells were seeded into six‐well plates at a density of 5 × 10^5^ cells/well and allowed to grow to confluency for 24 h. Next, a scratch was created across the cell layer using a sterile pipette tip. The plates were then gently washed with PBS and the culture medium was replaced with fresh serum‐free medium. Images were captured under an Olympus light microscope. Next, shRNA fragments were transfected into the cells and incubated for 4–6 h, followed by replacement with complete medium. Images were captured at the same position after 24 h of incubation. The area of the healed wound was measured using ImageJ software.

### 2.11 CCK‐8 cell metabolic colorimetric test

The CCK‐8 colorimetric was used to observe the change of cell proliferation. Single‐cell suspensions were seeded into 96‐well plates (100 μL/well). After transfection, the medium was replaced with complete medium and the plates were incubated at 37°C in a 5% CO_2_ atmosphere for 0, 24, 48, and 72 h. Next, 10 μL of CCK‐8 solution (Beyotime) was added to each well and incubated for an additional 4 h. The optical density (OD) of each well at 450 nm was measured using a multifunctional microplate reader (SYNERGY LX, BioTek).

### 2.12 Flow cytometry

For Annexin V and PI staining, 1 mL of 0.25% trypsin was used to create single‐cell suspensions (1 × 10^5^ cells/mL) that were placed in a flow tube. Next, the cells were washed twice with PBS, and then centrifuged at 1000 rpm for 5 min. The supernatant was discarded, and the cells were resuspended in 500 μL of binding buffer. The cells were then stained with 5 μL of Annexin V‐FITC and 10 μL of PI. For detection of CD8+ T cells, the cells were incubated with FITC anti‐human CD8a antibody (Elabscience, # E‐AB‐F1110C), and then analyzed with a flow cytometer (CytoFLEX S, Beckman Coulter).

### 2.13 Mice

Female nude mice (6–7 weeks old, weight: 20 ± 2 g) were purchased from the Experimental Animal Center of Southern Medical University. The mice were subcutaneously (s.c.) injected with either stable CNE2 or 5‐8F cells with a cell count of 5 × 10^6^. The weights of the mice and their tumor volumes were measured every 2 days. Vernier calipers were used to calculate the tumor volumes according to the following formula: (length × width^2^)/2. To observe the effect of B7‐H3 and 4‐1BB on tumor formation in nude mice in vivo, the mice were s.c. injected with a luc stable CNE2 cell line. After the mice developed tumors, they were intraperitoneally injected with 5 mg/kg anti‐4‐1BB (Abcam, #ab252559) or anti‐IgG (Abcam, # ab133470) twice a week, and then used for in vivo imaging experiments.

### 2.14 Histologic analysis of xenograft sections

NPC xenograft samples were fixed with 4% paraformaldehyde for 24 h and embedded in paraffin. The paraffin‐embedded section (3 μm) was stained with hematoxylin–eosin (H&E) for morphological assessment.

### 2.15 Immunohistochemistry

Paraffin‐embedded sections were dewaxed and rehydrated, and antigen retrieval was performed followed by antigen blocking. The paraffin‐embedded sections were then incubated with anti‐human Ki67 (Abcam, #ab16667, 1:200), anti‐human‐E‐cadherin (Abcam, #ab15148, 1:30), and anti‐human‐Bax (Abcam, #ab32503, 1:250) at 4°C overnight, and subsequently incubated with an RP‐linked‐goat‐anti‐rabbit IgG secondary antibody (Abcam, #ab6721,1:1000). DAB (#P0203, Beyotime) was used for color rendering.

### 2.16 In vivo imaging system

The tumorigenic mice were intraperitoneally injected with 150 µL of 30 mg/mL d‐Luciferin sodium fluorescein substrate (MCE, #HY‐12591). After 3–5 min, the mice were anesthetized, placed in a dark box platform, and the growth of the subcutaneous transplanted tumors was observed using a small animal in vivo imaging system (NightOWL Ⅱ LB983, Berthold).

### 2.17 Terminal deoxynucleotidyl transferase‐mediated dUTP nick end labeling (TUNEL) assay

Cell apoptosis was detected by the TUNEL assay. Terminal deoxynucleotidyl transferase biotin‐dUTP nick end labeling positive cells in tumor tissue sections from CNE‐2 mice were detected using a Cy3 TUNEL Assay Apoptosis Detection Kit (Solarbio, #T2195) and counterstained with nuclear DAPI staining (Solarbio, #C0065), following the manufacturer's protocol. The areas of TUNEL‐positive cells and DAPI‐positive cells in the total tumor section area were quantified using images obtained from a fluorescence microscope.

### 2.18 Enzyme‐linked immunosorbent assay (ELISA)

The levels of IFN‐γ and tumor necrosis factor‐α (TNF‐α) expression in the co‐culture system were detected using a Human IFN‐γ ELISA Kit (Elabscience, #E‐EL‐H0108c) and Human TNF‐a ELISA Kit (Elabscience, #E‐EL‐H0109c), respectively. The cells were pretreated and co‐cultured as described above. The levels of IFN‐γ and TNF‐α in mouse serum were detected using a mouse INF‐γ ELISA Kit (Elabscience, #E‐EL‐M0048c) and mouse TNF‐a ELISA Kit (Elabscience, #E‐EL‐M3063), respectively. The optical density (OD) at 450 nm was measured using a multifunctional microplate reader according to the manufacturer's instructions.

### 2.19 Statistical analysis

Statistical analysis and mapping were performed using Prism v8.0 software (GraphPad).

Data from different treatment groups were analyzed using the unpaired *t*‐test or one‐way analysis of variance. A *p* < .05 was considered to be statistically significant.

## 3 RESULTS

### 3.1 B7‐H3 suppression inhibited the proliferation and promoted the apoptosis of NPC cells

**Figure 1:**
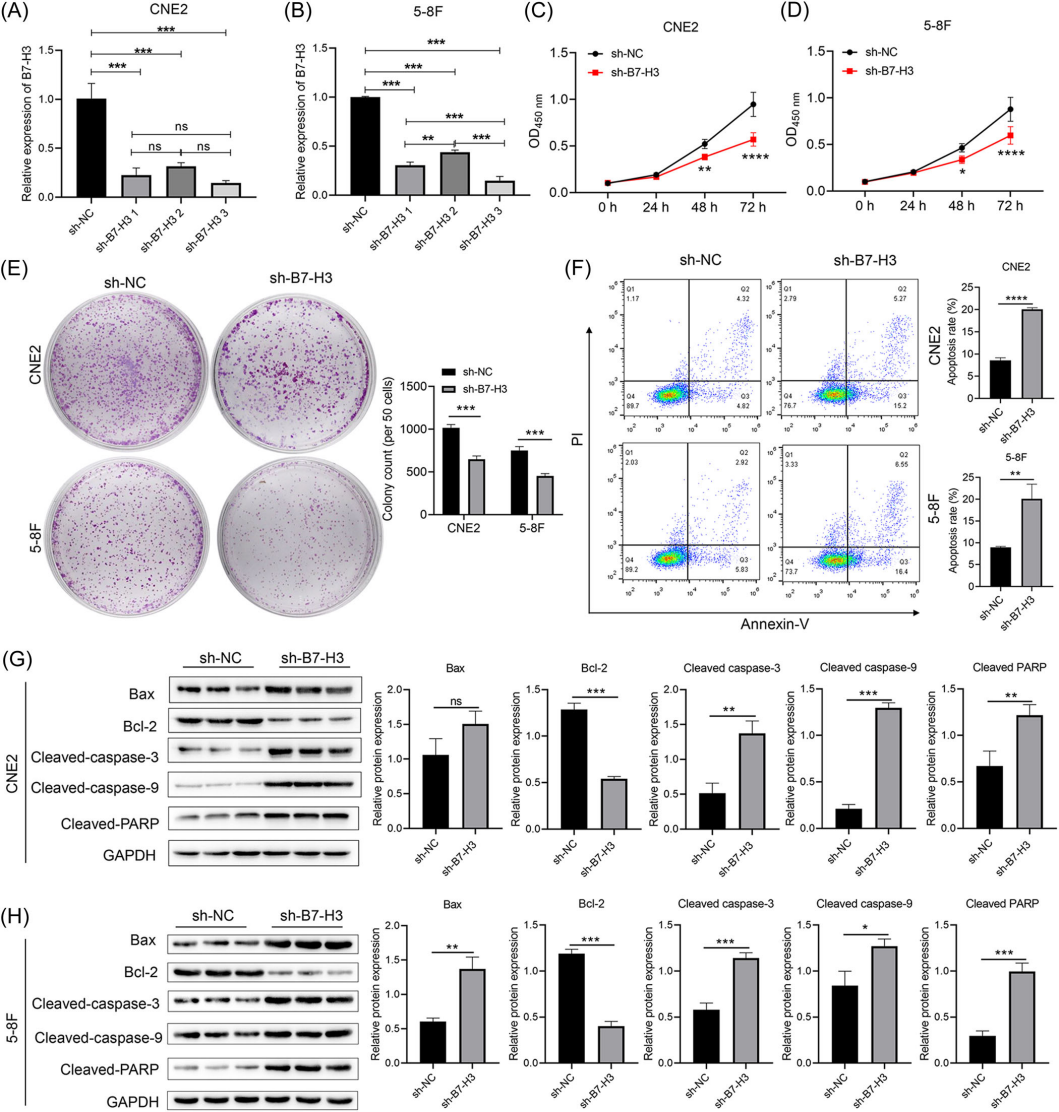
B7‐H3 suppression inhibited the proliferation and promoted the apoptosis of NPC cells. (A, B) B7‐H3 expression in CNE2 (A) and 5‐8F (B) cells transfected with sh‐B7‐H3‐RNA1, sh‐B7‐H3‐RNA2, and sh‐B7‐H3‐RNA3 was detected by real‐time quantitative PCR analysis. (C, D) The proliferation abilities of CNE2 (C) and 5‐8F cells (D) after transfection with sh‐B7‐H3 or sh‐NC were demonstrated by the CCK8 assay. (E, F) Colony formation ability of CNE2 and 5‐8F cells cultured after transfection with sh‐B7‐H3 or sh‐NC, as determined by the colony formation assay. (F) Statistical analysis of NPC cell apoptosis after transfection with sh‐B7‐H3‐RNA or the sh‐NC. (G, H) Western blot analysis showing the levels of Bax, Bcl2, cleaved‐caspase 3, cleaved caspase 9, and cleaved‐PARP in the indicated groups. *p < .05, **p < .01, ***p < .001, ****p < .0001. ns, no significance.

To investigate the effect of B7‐H3 on NPC cell behavior, we used RT‐qPCR and western blot analysis to screen for the most effective B7‐H3 shRNA (Figure 1A,B and Figure S1), and our data showed that sh‐B7‐H3‐RNA3 produced the greatest reduction in B7‐H3 mRNA and protein levels. CCK‐8 assay results (Figure 1C,D) showed that inhibition of B7‐H3 gene expression significantly reduced the proliferative ability of NPC cells, and reduced NPC cell colony numbers after sh‐B7‐H3‐RNA transfection proved that B7‐H3 reduced the proliferation of NPC cells (Figure 1E). These results suggested that the proliferative ability of the two NPC cell lines was significantly decreased after transfection with B7‐H3 shRNA when compared to the sh‐NC group.

To investigate the effect of B7‐H3 on NPC cell apoptosis, PI and Annexin‐V staining were performed on the cells, which were then analyzed by flow cytometry. The results showed that the total apoptosis rates (the sum of early apoptotic and late apoptotic cells) of CNE2 and 5‐8F NPC cells transfected with B7‐H3 shRNA were significantly higher than those in the sh‐NC group (Figure 1F). In addition, the expression levels of apoptosis‐related proteins were also detected. As shown in Figure 1G,H and Figure S2, after transfection with B7‐H3 shRNA, the levels of intracellular proapoptotic proteins, Bax, Cleaved caspase‐3, Cleaved caspase‐9, and Cleaved PARP, were significantly increased, while the levels of antiapoptotic protein, Bcl‐2, and the precursor protein levels of Caspase‐3, Caspase‐9, and PARP were significantly decreased. This indicated that B7‐H3 knockdown could significantly promote NPC cell apoptosis.

### 3.2 B7‐H3 suppression reduced the invasion, migration, and epithelial‐to‐mesenchymal transition (EMT) of NPC cells

**Figure 2:**
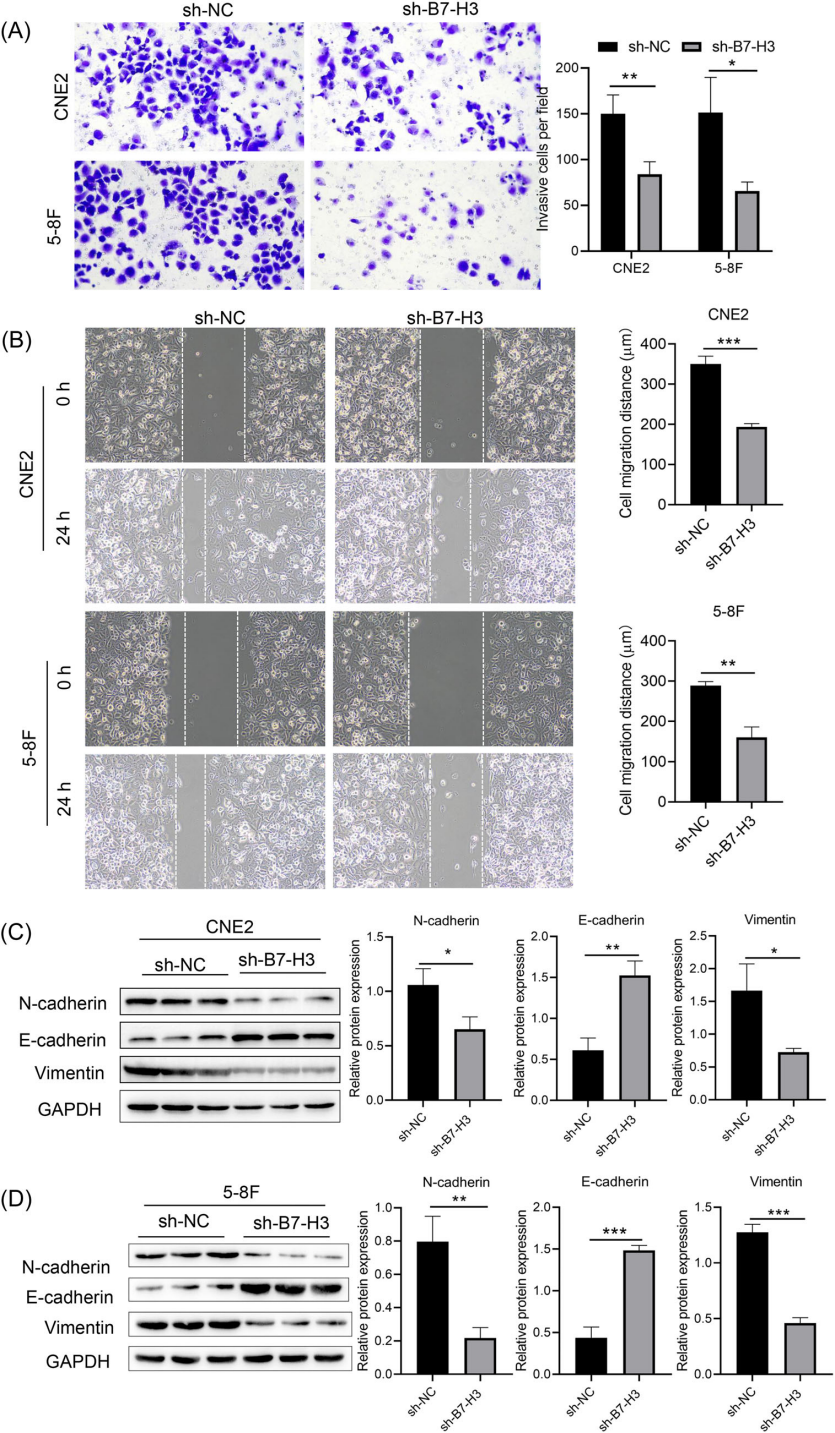
B7‐H3 suppression reduced the invasion, migration, and epithelial‐to‐mesenchymal transition abilities of NPC cells. (A) Representative images and statistical analysis of Transwell assays, *p < .05, **p < .01. (B) Representative images and statistical analysis of scratch assays, **p < .01, ***p < .001. (C, D) Western blot analysis showing the levels of N‐cadherin, E‐cadherin, and vimentin in sh‐B7‐H3 and sh‐NC transfected CNE2 (C) and 5‐8 F (D) cells. *p < .05, **p < .01, ***p < .001.

We next sought to determine how B7‐H3 influences NPC cell invasion and migration, which have a significant impact on the prognosis of patients. Transwell assays were performed to evaluate the invasion ability of NPC cells. The numbers of invasive cells in the sh‐B7‐H3 transfected NPC cell groups were significantly lower than those in the sh‐NC transfected NPC cell groups (Figure 2A). Scratch assays were also performed to detect cell migration ability. When compared with the sh‐NC group, the scratch areas of the two NPC cell lines transfected with B7‐H3 shRNA were significantly larger at 24 h after creating a scratch (Figure 2B).

EMT refers to the transformation of epithelium into interstitial cells, which gives cells the ability to invade and metastasize. It also induces stem cell characteristics, reduces apoptosis and senescence, and promotes immune suppression, which plays a key role in the development of cancer.^10^ We performed western blot studies to detect the levels of several proteins related to EMT, including vimentin, E‐cadherin, and N‐cadherin. The results showed that after transfection with B7‐H3 shRNA, the expression levels of mesenchymal cell‐characterizing proteins, N‐cadherin and Vimentin, were significantly decreased, while expression of the epithelial cell‐characterizing protein, E‐cadherin, was increased, indicating that B7‐H3 knockdown could significantly inhibit the occurrence of EMT in NPC cells (Figure 2C,D).

### 3.3 Stable knockdown of B7‐H3 inhibited tumor growth in vivo

**Figure 3:**
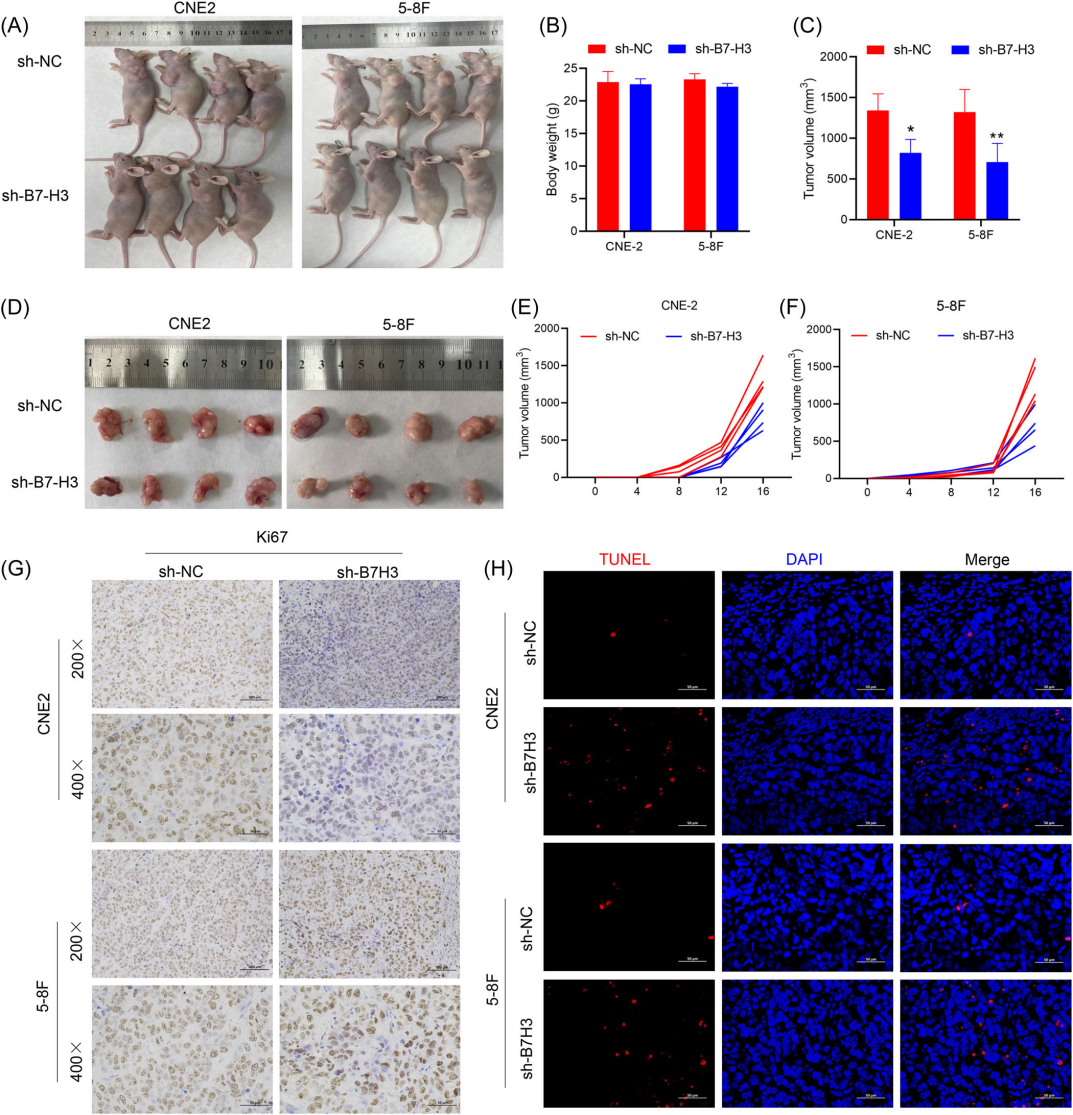
Stable knockdown of B7‐H3 inhibited tumor growth in vivo. (A) Images of the tumor‐bearing nude mice in different treatment groups. (B) Body weights of the mice in different treatment groups. (C) Tumor volume was significantly decreased when B7‐H3 was downregulated. *p < .05, **p < .01. (D) Images of CNE2 and 5‐8F xenograft tumors with or without stable knockdown of B7‐H3. (E, F) The growth curves ofCNE2 and 5‐8F xenograft tumors with or without stable knockdown of B7‐H3. (G) immunohistochemistry staining of Ki67 in CNE2 and 5‐8F xenograft tumors with or without stable knockdown of B7‐H3. (H) Images of Tunel staining in CNE2 and 5‐8F xenograft tumors with or without stable knockdown of B7‐H3.

Nude mice are commonly used as animal models for studying tumor behavior in vivo. To further explore whether B7‐H3 suppression in NPC cells affected tumor growth in vivo, we constructed cell lines with stable knockdown of B7‐H3 expression and subcutaneously inoculated nude mice with 5 × 10^6^ stable sh‐NC‐transfected cells or stable sh‐B7‐H3‐transfected cells to create xenograft tumor models. Consistent with our previous in vitro experiments, injection of the sh‐B7‐H3‐RNA‐transfected CNE2 cells or 5‐8F cells into the xenograft mice resulted in a lower body weight, lower tumor burden, and slower tumor growth when compared to injection of the sh‐NC‐transfected NPC cells into xenograft mice (Figure 3A–F). When the tumor tissues were harvested and subjected to immunohistochemistry (IHC) staining, lower levels of Ki67 expression were observed on tumor cells in the sh‐B7‐H3‐transfected xenografts (Figure 3G). TUNEL staining was used to detect apoptotic cells in xenograft tissues in situ. Sh‐B7‐H3‐RNA‐interfered xenograft tissues showed higher apoptosis rates than sh‐NC‐RNA‐interfered xenograft tissues (Figure 3H). Collectively, these results indicated that downregulation of B7‐H3 expression on NPC cells significantly inhibited tumor growth in vivo.

### 3.4 Stable knockdown of B7‐H3 promoted apoptosis and inhibited EMT in vivo

**Figure 4:**
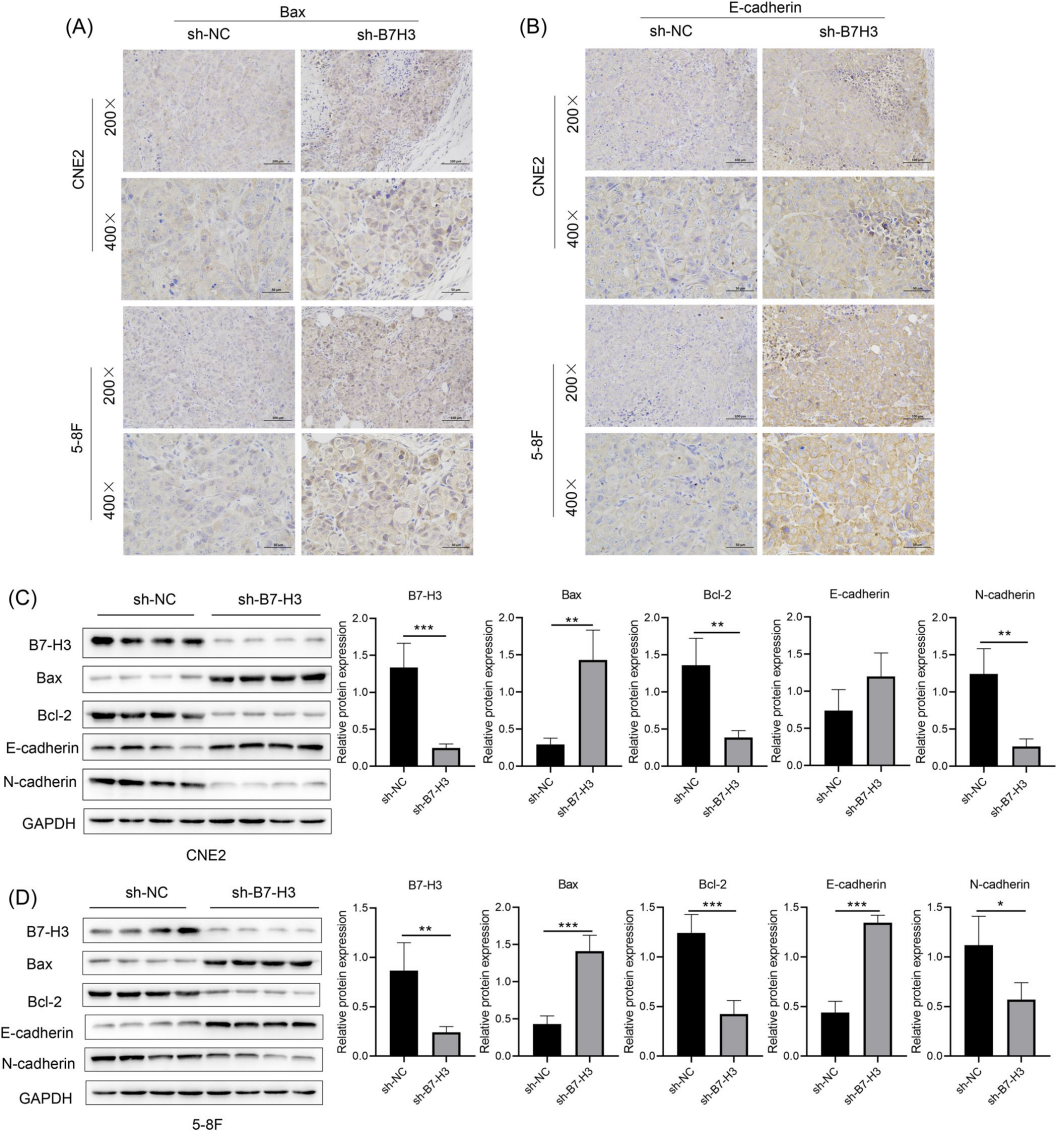
Stable knockdown of B7‐H3 promoted apoptosis and inhibited epithelial‐to‐mesenchymal transition in vivo. (A) immunohistochemistry (IHC) staining of Bax in CNE2 and 5‐8F xenograft tumors with or without stable knockdown of B7‐H3. (B) IHC staining of E‐cadherin in CNE2 and 5‐8F xenograft tumors with or without stable knockdown of B7‐H3. (C, D) Western blot analysis showing the levels of B7‐H3, Bax, Bcl2, N‐cadherin, and E‐cadherin in sh‐B7‐H3‐RNA and sh‐NC‐RNA transfected xenograft tumors. *p < .05, **p < .01, ***p < .001.

We next investigated the effects of B7‐H3 suppression on apoptosis and EMT in xenografts tissues. IHC staining showed an upregulation of Bax and E‐cadherin expression in sh‐B7‐H3‐RNA‐transfected tissues (Figure 4A,B), which indicated a higher apoptosis rate and less EMT phenomena. Furthermore, the total protein was extracted from the xenograft tissues and analyzed by western blot analysis. The results proved that B7‐H3 expression was significantly downregulated in the sh‐B7‐H3‐RNA‐transfected tissues. Moreover, expression of the proapoptotic protein, Bax, was upregulated in sh‐B7‐H3‐interfered tissues while expression of the antiapoptotic protein, Bcl‐2, was downregulated. Moreover, the expression levels of N‐cadherin were significantly decreased, while the expression levels of E‐cadherin were increased (Figure 4C,D), indicating that B7‐H3 knockdown could significantly promote apoptosis and inhibit the occurrence of EMT in vivo.

### 3.5 B7‐H3 inhibited tumor cell apoptosis by interacting with 4‐1BB on the surface of CD8+ T cells in vitro

**Figure 5:**
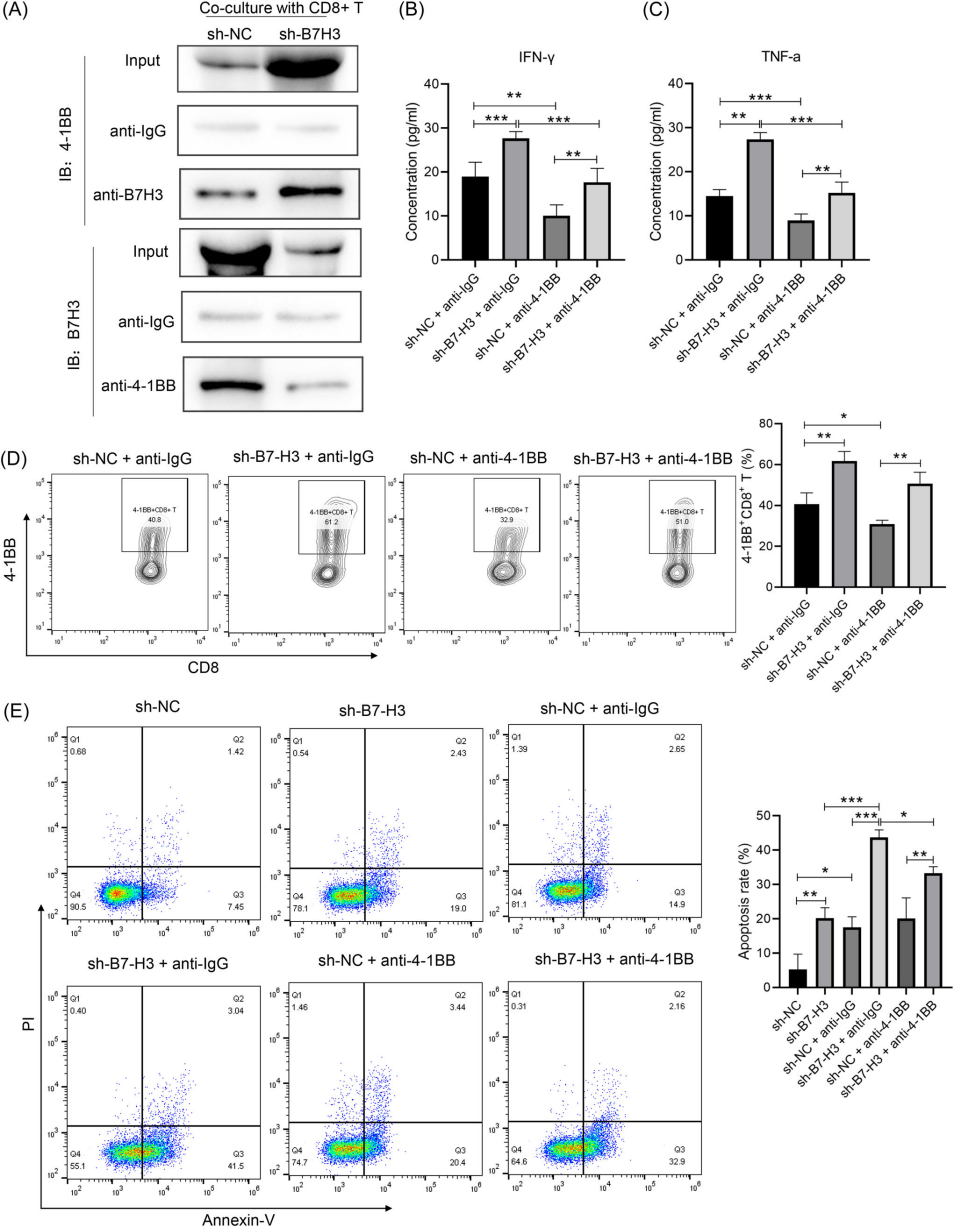
B7‐H3 inhibited tumor cell apoptosis by interacting with 4‐1BB on the surface of CD8+ T cells in vitro. (A) Co‐immunoprecipitation assays were performed using cell lysates from the CNE2 and CD8+ TIL co‐culture system. Immunoprecipitation was performed using a B7‐H3/4‐1BB antibody and results were analyzed by western blot analysis. (B, C) Supernatants from the co‐culture system were used for detection of interferon‐γ (B) and tumor necrosis factor‐α (C) via enzyme‐linked immunosorbent assay, **p < .01, ***p < .001. (D) Statistical analysis of CD8+4‐1BB+T cells in the coculture systems from different treatment groups as detected by flow cytometry, *p < .05, **p < .01. (E) Statistical analysis of apoptotic CNE2 cells in the coculture systems from different treatment groups, *p < .05, **p < .01, ***p < .001.

CD8+ T cells are generally considered as a homogeneous cell population. However, cytotoxic CD8+ T cells exhibit exceptional cytotoxic activity and efficiently kill tumor cells and cells harboring intracellular pathogens. Functionally, CD8+ T cells are characterized by high levels of perforin, granzyme B, IFN‐γ, and tumor necrosis factor (TNF‐α).^11^ Interestingly, B7‐H3 was predicted to interact with 4‐1BB, a cell receptor expressed on CD8+ tumor‐infiltrating lymphocytes (TILs). In our experiment, we designed a co‐culture system and used it to study the function of CD8+ T cells. CD8+ TILs were isolated from NPC tissues using CD8+ magnetic beads. The isolated CD8+ TILs were then co‐cultured with CNE2 cells. We collected cells from the co‐culture system and used them for co‐IP assays, and the results showed that B7‐H3 could interact with 4‐1BB (Figure 5A). Next, CD8+ TIL cells were pretreated with anti‐4‐1BB or anti‐IgG antibody, and subsequently co‐cultured with sh‐NC or sh‐B7‐H3‐transfected CNE2 cells. When compared with the sh‐NC group, the expression levels of IFN‐γ and TNF‐α in the co‐culture system were increased by B7‐H3 knockdown in CNE2 cells. However, after blocking 4‐1BB expression on the surface of CD8+ TIL cells, the expression levels of IFN‐γ and TNF‐α in the co‐culture system decreased (Figure 5B,C), suggesting that 4‐1BB inhibition could enhance the cytotoxic activity of CD8+ T cells. Flow cytometry was used to determine the proportion of CD8+4‐1BB+ TIL cells. As shown in Figure 5D, knockdown of B7‐H3 gene expression increased the proportion of 4‐1BB+CD8+ TIL cells, and blocking 4‐1BB reduced the proportion of 4‐1BB+CD8+ TIL cells when CNE2 cells transfected with sh‐NC. This finding suggested that B7‐H3 inhibits the antitumor effect of 4‐1BB by reducing the proportion of 4‐1BB+CD8+ TIL cells. Finally, the apoptosis of CNE2 cells after blockage of 4‐1BB on CD8+ TIL was detected by flow cytometry. Figure 5E shows that when CNE2 cells were cultured single, B7‐H3 knockdown could upregulate the apoptosis of CNE2 cells compared with the sh‐NC group. And when co‐cultured with CD8+ TILs, the apoptosis of CNE cells was significantly further increased whether treatment with sh‐NC or sh‐B7‐H3. However, the apoptosis of CNE2 cells in the sh‐B7‐H3 group was decreased after blocking 4‐1BB expression on the surface of CD8+TIL cells. In addition, three most extensively studied immune checkpoints were also selected to detect. As shown in Figure S3, when compared with the sh‐NC group, the expression levels of CTLA‐4, PD‐1 and TIM‐3 in CD8+ TIL cells were decreased by B7‐H3 knockdown in CNE2 cells. However, after blocking 4‐1BB expression on the surface of CD8+ TIL cells, the expression levels of CTLA‐4, PD‐1 and TIM‐3 in CD8+ TIL cells increased whether CNE2 cells transfected with sh‐NC or sh‐B7‐H3. This suggests that B7‐H3 may be an immune checkpoint for NPC and serve to inhibit the antitumor effect of 4‐1BB, which further clarifies the mechanism by which B7‐H3 promotes tumor growth.

### 3.6 B7‐H3 inhibited tumor cell apoptosis by interacting with 4‐1BB on the surface of CD8+ T cells in vivo

**Figure 6:**
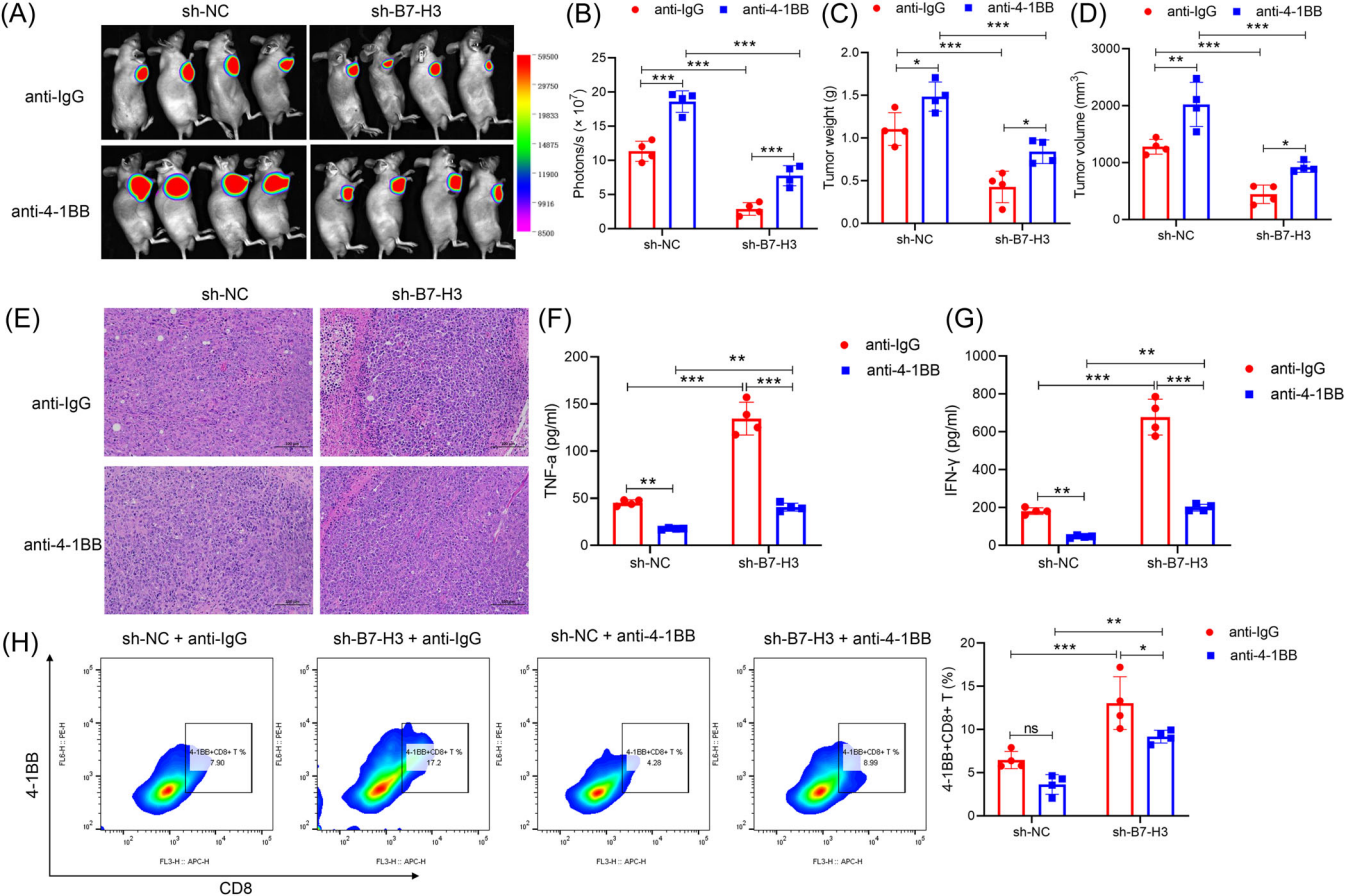
B7‐H3 inhibited tumor cell apoptosis by interacting with 4‐1BB on the surface of CD8+ T cells in vivo. (A–D) Mice transplanted with sh‐B7‐H3‐RNA or sh‐NC‐RNA transfected CNE2 cells were treated with anti‐4‐1BB or anti‐IgG antibody, and images obtained with an in vivo imaging system are shown. (A) The fluorescence intensity of xenograft tumors from each group of mice was recorded and statistically analyzed. (B) Tumor weight (C) and volume (D) were measured and statistically analyzed. (E) Images of hematoxylin–eosin‐stained xenograft tissue. (F, G) Mouse serum was used for detection of interferon‐γ (G) and tumor necrosis factor‐α (F) via enzyme‐linked immunosorbent assay. (H) A statistical analysis of CD8+4‐1BB+ T cells from xenograft tumors in mice. The cells were detected by flow cytometry.

We constructed a stable B7‐H3 knockout and luciferase gene 2 (Luc2)‐expressing NPC cell line and transplanted the cells subcutaneously into nude mice. We then intraperitoneally injected the nude mice with anti‐IgG or anti‐4‐1BB twice a week, respectively. When the tumors reached an appropriate size, each mouse was intraperitoneally injected with fluorescein substrate; after which, the mice were anesthetized and placed in a dark box platform to observe the growth of their tumors using an in vivo imaging system. Figure 6A–D shows that when compared with the control group, the volumes, weights, and luminescence intensities of the xenograft tumors with B7‐H3 knockdown were significantly decreased. These effects could be mitigated by use of an anti‐4‐1BB antibody, which again indicated that blocking 4‐1BB could promote the growth of NPC in vivo. H&E staining showed there were fewer necrotic areas in the tumor tissues when B7‐H3 gene expression was downregulated, and anti‐4‐1BB injection further ameliorated the necrotic phenomenon in tumor tissues (Figure 6E). In addition, the percentages of 4‐1BB+CD8+ T cells were significantly increased, and the levels of IFN‐γ and TNF‐a in peripheral blood serum were significantly increased when B7‐H3 expression was inhibited, and blocking 4‐1BB expression inhibited those effects. (Figure 6F–H). These results suggested that B7‐H3 could interact with 4‐1BB to promote NPC development.

## 4 DISCUSSION

Our study showed that B7‐H3, an important regulator of tumor immunity, plays a key role in NPC progression. In addition, B7‐H3 expression could correlate with 4‐1BB expression on CD8+ T cells, and inhibit the cytotoxic effect of CD8+ T cells on NPC cells.

NPC is well known for its high invasive ability. However, the molecular mechanisms that regulate the invasion and metastasis of NPC cells remain unclear and effective therapeutic strategies are still lacking. Immunotherapy has become a recent research hotspot for recurrent or metastatic NPC, and numerous clinical studies have been carried out in this area with the goal of prolonging the survival time of patients. Immunotherapy can effectively improve the immunosuppressive microenvironment and is widely used in the treatment of NPC. Inhibitory receptors expressed by T cells, antigen‐presenting cells (APC), and other cells, bind to their corresponding receptors and exert corresponding immune effects, which is collectively known as immune checkpoint.^12^ Among them, programmed death‐1 (PD‐1) and programmed death‐ligand1 (PD‐L1) are important targets for immunotherapy.^13^ In the tumor microenvironment, PD‐L1 that is expressed or overexpressed on the surface of tumor cells binds to PD‐1 targets expressed on the surface of T cells, and thereby inhibits the normal function of T cells and weakens their ability to recognize and respond to tumor cells, leading to tumor immune escape and formation of an immunosuppressive microenvironment.^14^

B7‐H3 (CD276), a member of the B7 ligand family, has recently emerged as an attractive target for antibody‐based immunotherapy. Substantial evidence demonstrates that B7‐H3 contributes to proliferation, metastasis, invasion, reduced apoptosis, and epithelial‐mesenchymal transition (EMT) in various types of tumor cells. Previous studies^15^, ^16^ showed that B7‐H3 promotes cancer cell invasion and migration by targeting EMT progression in hepatocellular carcinoma. Zhang et al.^17^ demonstrated that B7‐H3 promotes the proliferation and inhibits the apoptosis of ovarian cancer cells. Therefore, the role of B7‐H3 in cancer development can be of great importance. In this study, we investigated the biological functions of B7‐H3 in NPC by knocking down B7‐H3 expression in NPC cells. We found that when compared to a negative control group, downregulation of B7‐H3 in NPC cells significantly suppressed cell invasion, migration, proliferation, and EMT progression, but increased apoptosis, which proved that B7‐H3 plays an important role in NPC progression.

B7‐H3 directly affects tumor behavior, and our study showed that B7‐H3 participates in tumor immune evasion and influences a tumor's immune response by acting as an immune checkpoint for tumor cells. Du et al.^9^ reported encouraging results regarding the antitumor effect of novel CAR‐T cells loaded with B7‐H3 antibody, and that CAR‐T cells expressing 4‐1BB significantly reinforced the antitumor effect and reduced the expression levels of PD‐1 in CD8+ T cells. The initial function of 4‐1BB was found to be the activation of the killing ability of T cells and an induction of lymphocyte activation. The expression of 4‐1BB, an inducible expression receptor, can be detected on the surface of T cells activated by the CD3 monoclonal antibody, but not on the surface of resting T cells. While the levels of 4‐1BB expression on CD8+ and CD4+ T cells are similar, the mechanism of action of 4‐1BB on the two cell types is different. Numerous studies have investigated the regulatory effect of 4‐1BB on CD8+ T cells. Mihiko et al.^18^ found that 4‐1BB expression promotes the proliferation of CD8+ tumor‐infiltrating lymphocytes (TILs) in triple‐negative breast cancer (TNBC). Treatment with an agonistic anti‐4‐1BB antibody significantly enhances the cytotoxicity of CD8+ TILs, and provides a new approach for TNBC immunotherapy. Co‐stimulatory signals produced by 41BB promote the proliferation of both CD4+ and CD8+ T cells, but preferentially affect CD8+ T cells.^19^ A previous study reported that although the ratio of CD4+/CD8+ T cells was not significantly changed in 4‐1BB−/− and 4‐1BBL−/− mouse models, the numbers of CD8+ T cells and the immune response were decreased in the 4‐1BB−/− mouse models due to the decreased proliferation of naive T cells. Many studies have shown that 4‐1BB co‐stimulatory molecules promote cell activation and proliferation by activating the p38, JNK, NF‐κB, and other signaling pathways.^20^ The 4‐1BB co‐stimulatory molecule can not only promote the proliferation of T cells but also induce T cells to differentiate into central memory T cells, thereby alleviating T cell incompetence and failure.^21^

It has been speculated that B7‐H3 can interact with 4‐1BB, which is an inducible co‐stimulatory receptor expressed on activated T and NK cells.^22^ To investigate the effects of B7‐H3, 4‐1BB, and their interaction on NPC development and tumor immunity, we conducted further experiments. Our results indicated that when B7‐H3 was downregulated, the numbers of 4‐1BB+CD8+ TIL cells and the levels of pro‐inflammatory cytokines were significantly increased, while blocking 4‐1BB could mitigate those effects both in vivo and in vitro. This confirmed that B7‐H3 might interact with 4‐1BB to regulate CD8+ T cell exhaustion and contribute to NPC progression.

Our study does have some limitations, for example: (1) The specific signaling pathways were not investigated. (2) Due to laboratory conditions, only CNE‐2 and 5‐8 F cells were used to perform tumor formation assays in nude mice. (3) When studying B7‐H3 and the tumor microenvironment, we focused mostly on the function of CD8+ TILs, and the relationship between B7‐H3 and other immune cells, tumor‐associated fibroblasts, and endothelial cells, could have been further investigated. Therefore, more research is needed to explore the development of targeted therapeutics for this disease.

In conclusion, our study provides insights into how overexpression of B7‐H3 in NPC cells creates a milieu of pro‐metastatic and growth‐promoting factors that promote tumor growth and distant metastasis. Moreover, B7‐H3 promotes tumor immune evasion by inhibiting CD8+ T cells via interaction with 4‐1BB. These results shed light on the possibility of B7‐H3 and/or its associated molecules serving as therapeutic targets for NPC.

## AUTHOR CONTRIBUTIONS

Xiaowen Zhang, Wenjing Liao, Guangui Chen, and Zhaoen Ma conceived and designed the experiments. Zhaoen Ma, Gui Chen, Hao Li, and Saixuan Yang performed the experiments and Wenjing Liao analyzed the data. Zhaoen Ma, Wenjing Liao, Saixuan Yang, and Yali Xu wrote the paper. All authors revised and approved the manuscript.

## CONFLICT OF INTEREST STATEMENT

The authors declare no conflict of interest.

## ETHICS STATEMENT

All NPC patients were recruited according to the protocol that was approved by the clinical Ethics Committees of the First Affiliated Hospital of Guangzhou Medical University. This study adhered to the tenets of the Declaration of Helsinki. Written informed consent was obtained from all participants.

All animal experiments were carried out strictly according to the guidelines of the Animal Centre of the First Affiliated Hospital of Guangzhou Medical University, and the experimental procedures were approved by the Laboratory Animal Ethics Committee of the First Affiliated Hospital of Guangzhou Medical University (Approval No. 20230504).

## Supporting information

Supporting information.

Supporting information.

Supporting information.

Supporting information.

## Data Availability

All data in this study are available from the corresponding author with a reasonable request.
