# Supplementary figures and images for: B7‐H3 promotes nasopharyngeal carcinoma progression by regulating CD8+ T cell exhaustion
Source: Immun Inflamm Dis. 2024 Sep 13;12(9):e70005. doi: 10.1002/iid3.70005 (PMC11393430; doi:10.1002/iid3.70005)

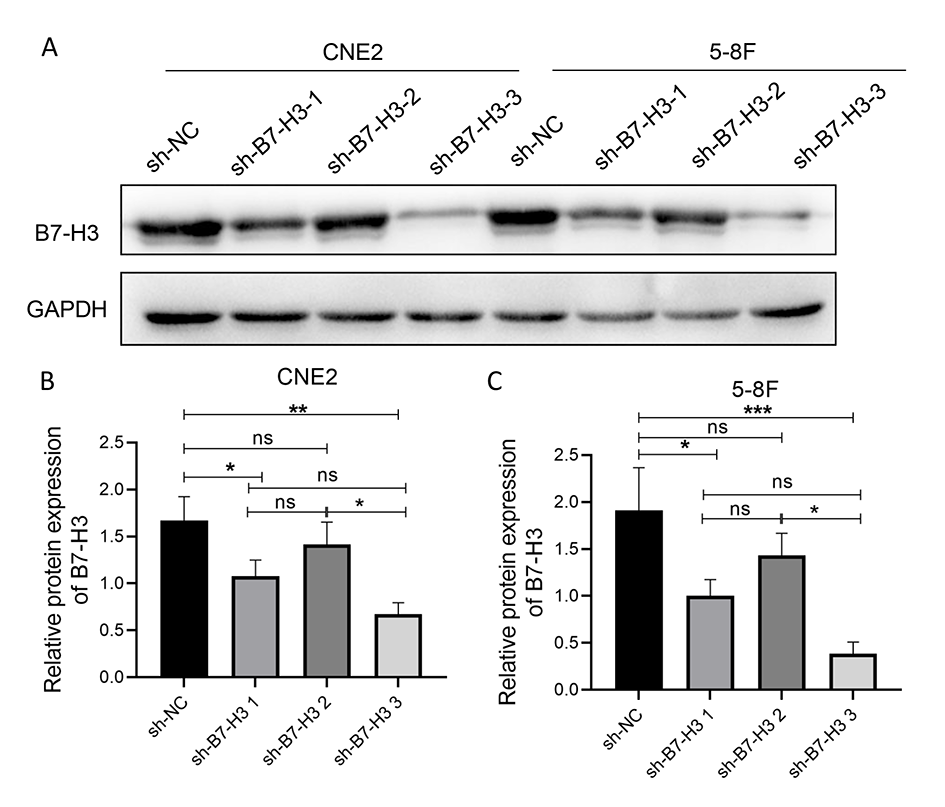

Supplement: Supplementary file 1 — Supporting information. [file IID3-12-e70005-s001.tif]

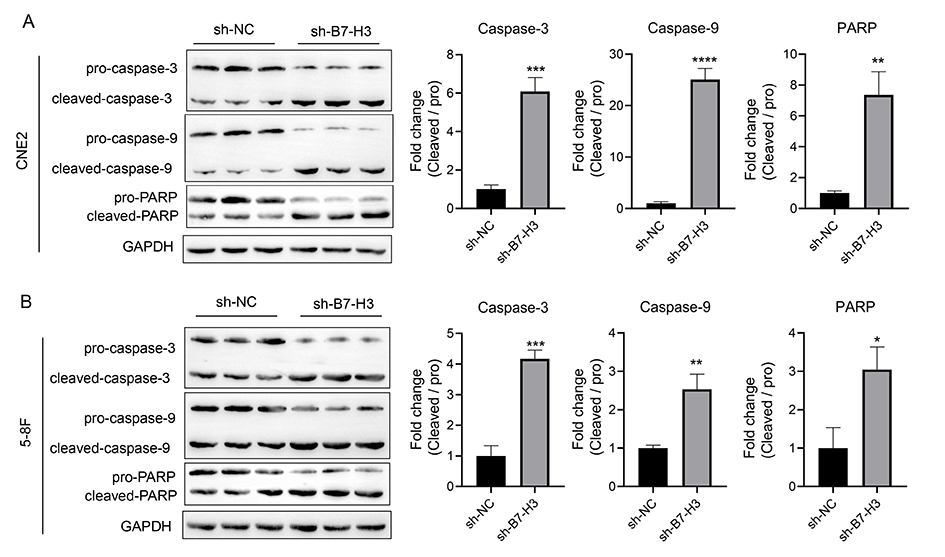

Supplement: Supplementary file 2 — Supporting information. [file IID3-12-e70005-s004.tif]

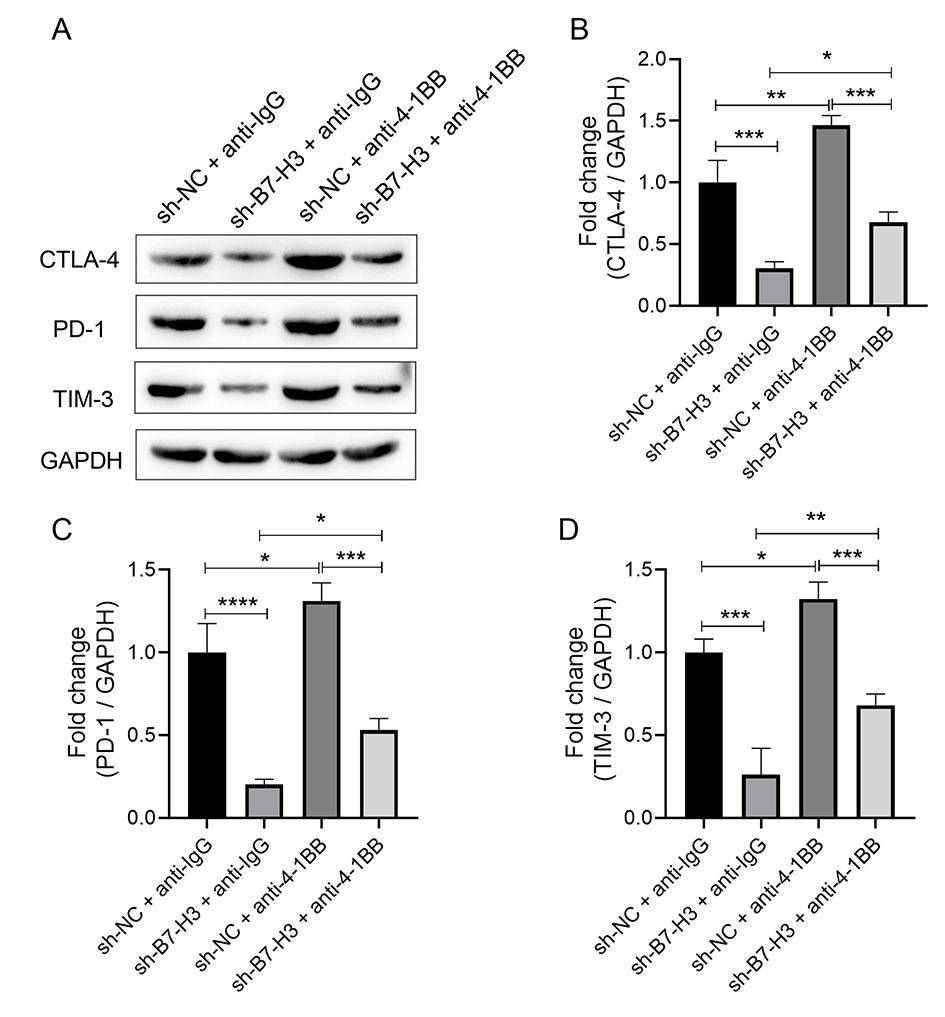

Supplement: Supplementary file 3 — Supporting information. [file IID3-12-e70005-s003.tif]
