# Supplementary material for: B7‐H3 promotes nasopharyngeal carcinoma progression by regulating CD8+ T cell exhaustion
Source: Immun Inflamm Dis. 2024 Sep 13;12(9):e70005. doi: 10.1002/iid3.70005 (PMC11393430; doi:10.1002/iid3.70005)
Supplement: Supplementary file 4 — Supporting information. [file IID3-12-e70005-s002.docx]

Figurementary Figure 1. **Western blotting was used to screen the most interference efficient shRNA. (A)** Western blotting analysis of B7-H3 protein level in NPC cells transfected with sh-NC or sh-B7-H3. **(B)** Quantification of B7-H3 protein band (n▒=▒3). Data are presented as mean▒±▒standard deviation (SD). *P▒<▒0.05, **P▒<▒0.01, ***P▒<▒0.001. Ns: no significance.

Figurementary Figure 2. **The levels of intracellular apoptosis-associated proteins were detected in NPC cells after transfected with sh-NC or sh-B7-H3. (A-B)** Western blotting analysis of Caspase-3, Caspase-9 and PARP protein levels in NPC cells transfected with sh-NC or sh-B7-H3. n▒=▒3. Data are presented as mean▒±▒standard deviation (SD). *P▒<▒0.05, **P▒<▒0.01, ***P▒<▒0.001.

**Supplementary Figure 3. (A)** Western blotting analysis of CTLA-4, PD-1 and TIM-3 protein levels in CD8▒+▒TIL cells from the co-culture systems. **(B-D)** Statistical analysis of CTLA-4, PD-1 and TIM-3 protein levels from different treatment groups. Data are presented as mean▒±▒standard deviation (SD). *p▒<▒0.05, **p▒<▒0.01, ***p▒<▒0.001.
